# Potential of Immunotherapies in Treating Hematological Cancer-Infection Comorbidities—A Mathematical Modelling Approach

**DOI:** 10.3390/cancers13153789

**Published:** 2021-07-27

**Authors:** Johnny T. Ottesen, Morten Andersen

**Affiliations:** 1 Center for Mathematical Modeling-Human Health and Disease (COMMAND), Roskilde University, 4000 Roskilde, Denmark; moan@ruc.dk; 2 IMFUFA, Department of Science and Environment, Roskilde University, 4000 Roskilde, Denmark

**Keywords:** cancer-infection-immune coupling, cancer-infection comorbidity, immunotherapy, CAR T-cell therapy, mathematical modelling, in silico investigation, bi-stability, dormant state, the three E’s of immunoediting

## Abstract

**Simple Summary:**

The immune system protects the human body against threats such as emerging cancers or infections, e.g., COVID-19. Mutated malignant cells may in many cases be controlled by the immune system to be kept at an unnoticed low amount. However, a severe infection may compromise the immune system in controlling such malignant clones leading to escape and fatal cancer progression. A novel mechanism based computational model coupling cancer and infection to the adaptive immune system is presented and analyzed. The model pin-points important physiological mechanisms responsible for cancer progression and explains numerous medical observations. The progression of a cancers and the effects of treatments depend on cancer burden, the level of infection and on the efficiency of the adaptive immune system. The model exhibits bi-stability, i.e., gravitate towards one of two stable steady states: a harmless dormant state or a full-blown cancer-infection disease state. A borderline exists and if infection exceeds this for a sufficiently long period of time the cancer escapes. Early treatment is vital for remission and may control the cancer back into the stable dormant state. CAR T-cell immunotherapy is investigated by help of the model. The therapy significantly improves its efficacy in combination with antibiotics or immunomodulation.

**Abstract:**

Background: The immune system attacks threats like an emerging cancer or infections like COVID-19 but it also plays a role in dealing with autoimmune disease, e.g., inflammatory bowel diseases, and aging. Malignant cells may tend to be eradicated, to appraoch a dormant state or escape the immune system resulting in uncontrolled growth leading to cancer progression. If the immune system is busy fighting a cancer, a severe infection on top of it may compromise the immunoediting and the comorbidity may be too taxing for the immune system to control. Method: A novel mechanism based computational model coupling a cancer-infection development to the adaptive immune system is presented and analyzed. The model maps the outcome to the underlying physiological mechanisms and agree with numerous evidence based medical observations. Results and Conclusions: Progression of a cancer and the effect of treatments depend on the cancer size, the level of infection, and on the efficiency of the adaptive immune system. The model exhibits bi-stability, i.e., virtual patient trajectories gravitate towards one of two stable steady states: a dormant state or a full-blown cancer-infection disease state. An infectious threshold curve exists and if infection exceed this separatrix for sufficiently long time the cancer escapes. Thus, early treatment is vital for remission and severe infections may instigate cancer progression. CAR T-cell Immunotherapy may sufficiently control cancer progression back into a dormant state but the therapy significantly gains efficiency in combination with antibiotics or immunomodulation.

## 1. Introduction

Inflammation is a hallmark of cancer [1,2,3,4,5] and the purpose of the present work is to illuminate how the immune system bridges cancer-infection comorbidities [6]. This interaction is essential for the disease progression and in situ cancer progression [7,8]. The present work contributes to the understanding of the complexity of the principal dynamics of the cancer-infection coupling and to fertilize an ongoing debate on advancing existing clinical immunotherapies [6,9,10,11]. Inflammation related to chronic inflammatory bowel diseases, obesity, aging and smoking significantly increases the risk of several types of cancers [12,13,14,15]. For cancers of the blood forming system, such as the myeloprofilerative neoplasms (MPNs), the connection between disease development and chronic inflammation is an active research area [16,17,18,19,20] and diagnostics and treatments specifically targeting the inflammatory component of the disease are of interest to clinicians [21,22,23]. Hematopoietic stem cells are located in the bone marrow and gives rise to the various blood cells in the peripheral blood [24]. Hematopoietic stem cells interact through their microenvironment and via immunesurveillance by the adaptive immune system [4].

The well-known logistic growth model may be used to describe growth of single clones [25,26,27]. We partly adopt this approach for malignant and infectious cells and add effects instigated by the adaptive immune system. The effector cells of the adaptive immune system may be activated by malignant cells to defeat the pathogen specific malignant cells. Kuznetsov and Knott were the first to model this in a quantitative cancer-immune surveillance model in 1994 and 2001 [28,29]. The dynamics of the complex cancer-immune system became quantitatively better understood by Kuznetsov and Knott’s simple model and the principal mechanisms governing the dynamics became cemented as the theoretical foundation for understanding the cancer-immune interaction at systemic level. This theory is the core idea of immunotherapy. Inspired by Kuznetsov and Knott, many modeling papers on cancer-immune system have emerged, see for example [30,31,32,33,34]. The novelty of the present work is to address comorbidities in this context by presuming that the immune system has a limited capacity. This agrees with the decreasing potential hypothesis outlined in [35]. The immune system needs to balance responses to several simultaneous attacks, which may suggest multi-modality treatments. In the present study, we consider chimeric antigen receptor (CAR) T-cell immunotherapy [36,37]. Our model shows how the progression of cancer and the effect of treatments inherently depend on coupling and the levels of the cancer and infection through inflammation. Therefore, we investigate the selected treatments in combination with anti-inflammatory or immunomodulation treatment. We explore a threshold phenomenon where the levels of cancer and infection in combination determine whether immune surveillance can control the outbreak of the disease with or without treatment by immunotherapy. In order to reduce the complex dynamics of the model of the physiological system by solely focusing on the governing principal mechanisms, care is needed. We base our principal mechanisms on well established biological knowledge and show that these principal mechanisms alone may explain multiple clinical observations. Our modeling approach is best described by quoting both George Box, “All models are wrong, but some are useful” [38], and Albert Einstein, “keep it as simple as possible, but no simpler” (communicated by Roger Sessions in the New York Times, 8 January 1950) [39].

## 2. Materials and Methods

We propose a model describing cancer and infection growth as logistic in the absence of the adaptive immune response [27]. Such models are well documented and have been used to fit various in vitro data as well as in vivo data [26]. In the presence of the adaptive immune response, additional inhibitory terms are added to the model equations. These terms are proportional to the product of the amount of pathogens and effector cells (both normalized relative to the respective carrying capacities) multiplied by a reaction rate and the probability for the pathogens to subsequently become eliminated. The hitherto uncoupled cancer and infection equations are coupled by the adaptive immune system. In order to reduce the large complexity of the immune system, we lump all the inhibitory feedback mechanisms regulating the activation of pathogen specific effector T-cells into one mechanism: the T-cell regulatory pathway. Specifically, we represent this T-cell regulatory pathway by a lumped compartment with a constant production rate. This choice is conservative and establishes a bottleneck limiting the instantaneous capacity of activating pathogen specific effector T-cells. Thus, if the host needs to allocate resources to fight a pathogen, these resources are not available to fight other threats. Such coupling effect via the adaptive immune response becomes pronounced for a large amount of pathogens, while it may be insignificant for small amounts. This presumption is generally termed the decreasing potential hypothesis [35].

**Figure 1:**
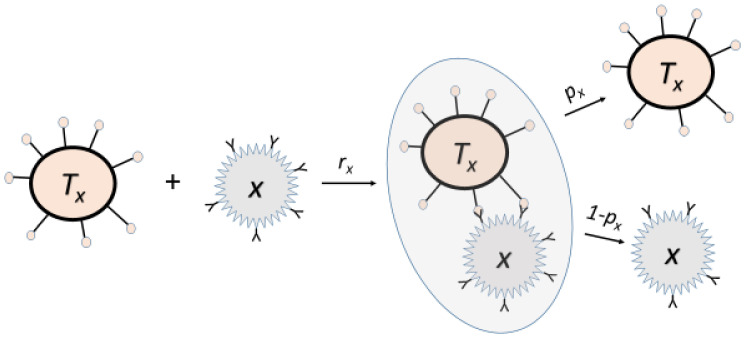
Naïve T-cells are activated by pathogens (including cancer cells) via dendrite cells to produce pathogen specific cytotoxic T-cells ($T_{x}$) by detecting the pathogens (*x*). The cytotoxic T-cells are eliminated naturally or removed from the pool of effector cells, with probability $1-p_{x}$ after binding to pathogens a number of times. The pathogens are eliminated with probability $p_{x}$.

The rate of change of cancer growth is assumed to consist of two terms; the first term is the aforementioned logistic growth, whereas the second term is an additional elimination enforced by the adaptive immune system according to the law of mass action. The adaptive immune response may be activated by pathogens—such as infections or malignant cells—and initiate a production of pathogen specific effector cells in order to eliminate the pathogens in the case of infection or malignant cells. We assume that the T-cell regulatory pathway activates the pathogen specific effector T-cells, i.e., cytotoxic T-cells. This regulatory pathway involves cytokines, signaling molecules, antigen presenting cells, regulatory T-cells and naïve T-cells. We associate the size of the cancer and the strength of the immune response with cell counts and we will use these phrases interchangeably. Furthermore, cancer and infection size will be relative to their carrying capacities, hence, a fraction between 0 and 1 corresponding to 0% and 100%. Generally, naïve T-cells are activated to produce cancer specific cytotoxic T-cells, $T_{x}$. Cancer specific effector T-cells bind to the cancer cells and initiate cancer cell apoptosis, with probability $p_{x}$. The activation of T-cells is mediated by antigen presenting cells, such as dendrite cells, detecting the cancerous cells while the B-cell also plays a role. The cytotoxic T-cells are eliminated naturally or may be depleted upon binding to cancer cells, with probability $1-p_{x}$ neglecting the possibility of reversible unbinding without effect. Several pathways regulate the depletion of activated cytotoxic T-cells [40,41]. In addition, exhaustion of individual cytotoxic T-cells is reported [42,43], which may be considered as removal of active cytotoxic T-cells from the pool of such. The result of these mechanisms is a reduction in the number of activated cytotoxic T-cells initiated by their own activity upon binding to the pathogens. Thus, we presume that T-cell depleting pathways are initiated upon T-cells binding to pathogens resulting in the removal of T-cells from the active cytotoxic T-cell pool with a small but non-vanishing probability. This hypothesis is further supported by [44,45,46,47]. The simplified mechanistic process underlying the elimination enforced by the adaptive immune system onto the cancerous cells is illustrated in Figure 1.

Similarly, an infectious development may be described by logistic growth with an explicit elimination added due to the adaptive immune system [48]. In absence of immune response, the amount of infectious pathogens would reach a plateau level in the carrying capacity. When introducing the adaptive immune modulation, the carrying capacity changes. A small modulation (corresponding to a few T-cells) reduces the plateau level correspondingly, while larger modulation (corresponding to high level of T-cells) reduces the plateau level to zero and thereby eliminates the infection. The naïve T-cells are activated to produce infection specific cytotoxic T-cells or effector cells, $T_{y}$. Such infection specific cytotoxic T-cells kill the infected cells with probability $p_{y}$. However, in addition to natural elimination, the effector cells may be removed with probability $1-p_{y}$ in a second order pathogen dependent process (according to the law of mass action). Thus, the two species, cancer and infection, may be described by similar equations.

(1a) $$\begin{array}{cc} x^{\prime } & =a_{x}x\left(1-\frac{x}{K_{x}}\right)-r_{x}p_{x}T_{x}x, \end{array}$$

(1b) $$\begin{array}{cc} y^{\prime } & =a_{y}y\left(1-\frac{y}{K_{y}}\right)-r_{y}p_{y}T_{y}y. \end{array}$$

Here $x^{\prime }$ is the rate of change of the number of cancerous cells, *x*. The growth rate is denoted $a_{x}$ and the intrinsic carrying capacity is denoted $K_{x}$. The last term represents the effect of the adaptive immune system. Here, $r_{x}$ is the per capita rate of binding between cancer specific cytotoxic T-cells and the cancer cells, $p_{x}$ is the probability that the cytotoxic T-cell initiates cancer cell apoptosis, while $T_{x}$ is the amount of cancer specific cytotoxic T-cells. Equation (1b) for the number of infected cells, *y*, is similar to Equation (1a). The key features of the model are the following: (i) sufficiently strong immune response may eradicate infectious pathogens in absence of cancers in agreement with experiences, (ii) the low disease co-existing stable steady state may be undetectable and low in infection as well as in malignant cells and (iii) a significant infection may instigate cancer escape in the model, which is in accordance with speculations by clinicians.

**Figure 2:**
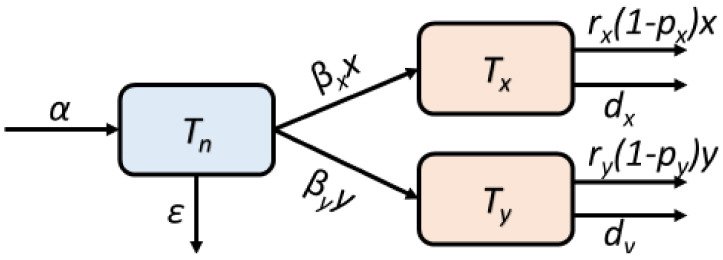
The T-cell regulatory pathway, which involves the naïve T-cells, is denoted $T_{n}$. The baseline production rate is denoted α and natural elimination rate ϵ. The pathogen specific cytotoxic T-cells are produced from this T-cell regulatory pathway. The amount of cancer cells is denoted by *x*, while that of infected cells is denoted by *y*. The amount of the cancer specific effector cells is denoted $T_{x}$ and is produced with the per capita rate $\beta_{x}x$ while the amount of the infection specific effector cells is denoted $T_{y}$ and is produced with the per capita rate $\beta_{y}y$. The effector cells have natural death rates $d_{x}$ and $d_{y}$, respectively. In addition, active cytotoxic T-cells may become inactivated or exhausted, which adds an extra removal from the pools of active cytotoxic T-cells. The removal per time is assumed proportional to the respective binding with cancer cells and infected cells multiplied by the probabilities $1-p_{x}$ and $1-p_{y}$, respectively.

The rate of change for the T-cell regulatory pathway ($T_{n}$) is provided by a baseline production rate, α, and natural elimination rate, ϵ, as well as terms describing the conversion of naïve T-cells into specific effector T-cells. The conversions are taken to be proportional to the respective load of pathogens. The cancer specific effector immune cells are produced proportional to the relative amount of cancer cells times the amount of naïve T-cells represented by the T-cell regulatory pathway. Moreover, they are eliminated with a constant natural death rate in addition to the probability of becoming inactivated or exhausted upon binding to cancer cells. Similarly, the infection specific effector cells are produced proportional to the relative amount of infected cells times the amount of naïve T-cells. They are eliminated with a constant natural death rate in addition to the probability of becoming inactivated or exhausted upon binding to infected cells. These processes are illustrated in Figure 2.

For the T-cell regulatory pathway, we have the following:

(2) $$T_{n}^{\prime }=\alpha -\beta_{x}xT_{n}-\beta_{y}yT_{n}-\epsilon T_{n}\;,$$

where $T_{n}$ denotes the T-cell regulatory pathway and $T_{n}^{\prime }$ denotes the rate of change of $T_{n}$, with the baseline production rate α and natural death rate ϵ. The per capita rates by which the cancer and infection stimulate the production of the respective specific effector cells are denoted by $\beta_{x}$ and $\beta_{y}$. For the amount of cancer specific effector immune cells, $T_{x}$, we have the following:

(3) $$T_{x}^{\prime }=\beta_{x}xT_{n}-r_{x}\left(1-p_{x}\right)T_{x}x-d_{x}T_{x}\;,$$

where $T_{x}^{\prime }$ denotes the rate of change of $T_{x}$ having a natural death rate $d_{x}$. Finally, the number of infection specific effector cells, $T_{y}$, with rate of change $T_{y}^{\prime }$ and natural death rate $d_{y}$ is provided by the following.

(4) $$T_{y}^{\prime }=\beta_{y}yT_{n}-r_{y}\left(1-p_{y}\right)T_{y}y-d_{y}T_{y}\;.$$

The presence of malignant cells and infected cells result in activation of the respective immune effector cells, which subsequently try to eliminate the cancer and infected cells. Hereby, the cancerous system and the infectious system are specifically coupled and share resources of the adaptive immune system. As a consequence, the size of the CD8+ T-cell pool is limited, e.g., during virus infections in agreement with [8,49,50,51].

In the following, we limit ourselves to consider cancer (with a time scale months-years) and infection (with a time scale weeks-years) with slow dynamics compared to the faster dynamics of the immune system (with a time scale hours-days). Thus, the slow manifold approximation may be legitimated by the use of singular perturbation theory [52,53], see Appendix A. This approximation results from letting the right-hand side of the three differential equations for the immune cells to vanish. Hence, we arrive at the following.

(5a) $$\begin{array}{cc} T_{n} & =\frac{\alpha }{\beta_{x}x+\beta_{y}y+\epsilon } \end{array}$$

(5b) $$\begin{array}{cc} T_{x} & =\frac{\beta_{x}x}{r_{x}\left(1-p_{x}\right)x+d_{x}}T_{n} \end{array}$$

(5c) $$\begin{array}{cc} T_{y} & =\frac{\beta_{y}y}{r_{y}\left(1-p_{y}\right)y+d_{y}}T_{n}\;. \end{array}$$

Inserting these equations into Equations (1a) and (1b), describing cancer and infection, and introducing dimensionless variables $X=\frac{x}{K_{x}}$, $Y=\frac{y}{K_{y}}$ and $\tau =a_{x}t$, we obtain the following reduced model

(6a) $$\begin{array}{cc} \frac{dX}{d\tau } & =X\left(1-X\right)-A_{1}\frac{X^{2}}{\left(A_{2}X+A_{3}Y+1\right)\left(A_{4}X+1\right)} \end{array}$$

(6b) $$\begin{array}{cc} \frac{dY}{d\tau } & =B_{0}Y\left(1-Y\right)-B_{1}\frac{Y^{2}}{\left(A_{2}X+A_{3}Y+1\right)\left(B_{4}Y+1\right)}\;. \end{array}$$

Equations (6), which coins the coupled *cancer-infection-immune model*, is investigated in the following: The parameters $A_{i}$ and $B_{i}$ consist of clusters of the original parameters, see Table 1. We emphasize that the system of equations in Equation (6) has seven parameters governing the progression of the coupled cancer-infection-immune system. This reduction in the number of parameters from the 14 physiological parameters in the original equations is advantageous. One may easily calculate the values of the clustered parameters from the physiological ones but not vice versa. The reduction in the number of governing parameters greatly advances the analysis of the model. The interpretation of the clustered parameters are as followes: $A_{4}$ and $B_{4}$ represent the ratio of the strengths of the respective second order elimination of effector cells to their natural death rates. $A_{2}$ and $A_{3}$ represent the ratio of the rate of production of the respective effector cells per capita to the natural death rates of their predecessor. $A_{1}$ and $B_{1}$ represent the product of the ratio of the baseline production of the predecessor to the growth rate of the pathogens with the ratio of the binding reaction rate multiplied by the probability of the pathogen being eliminated to the natural death rate of the effector cells (times $A_{2}$ and $A_{3}$, respectively). $B_{0}$ is the ratio between the intrinsic growth rate for infection and that for cancer.

The dynamics of the coupled cancer-infection-immune system in Equation (6) are quantitatively studied by in silico methods (using Matlab2020a) and the principal mechanisms governing the dynamics are qualified and interpreted. Simulations are made for MPNs, which often develops unnoticed over 20–40 years, while the characteristic times scale for cancer development is in years. However, the actual time scale does not affect the model outcome qualitatively, meaning that the results for at least non-solid tumors are similar except for the time scale. Where nothing else is emphasized, the default parameter values are taken from our previous estimates as described in Appendix A.

## 3. Results

### 3.1. General In Silico Dynamics and Disease Progression

The model encapsulates earlier model results for cancer progression without infection and infection without malignant cells. If no malignant cells are present, low or high burden infection may occur. If no infection exists, low or high cancer burden may occur (noticing that fully sterile environment is an idealization). The low-level burden and high-level burden are separated by threshold values. In this paper, we are interested in examining robust dynamics, i.e., allowing small perturbations of infections and malignant mutations. None of the aforementioned states with vanishing infection or malignant cells are stable. Particularly, the trivial state with no infection and no malignant cells is not stable. Thus, we focus on the co-existing states in the remaining part of the article.

**Figure 3:**
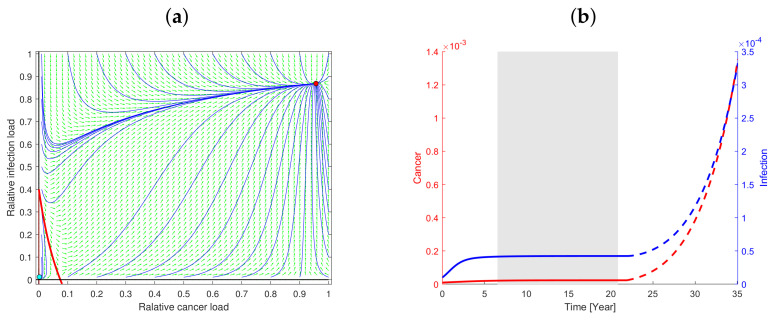
Cancer-infection-immune dynamics. Populations are normalized by their carrying capacities. (**a**) The phase plane with relative cancer size on first axis and relative infection burden on second axis. Circles, red for the full-blown disease state (to the upper right) and magenta for the dormant state (to the lower left) indicate the two stable co-existing steady states. Notice that the dormant state has very low but non-vanishing levels of malignant cells and infection. The solid blue curves are solution curves evolving over time toward one of the stable steady states. Green arrows indicate the flow direction at any point as these are tangent to the solution curves of Equation (6). The phase plane is divided into two regions by the red curve (the separatrix) depending on which stable steady state is approached. (**b**) The red curve is cancer progression while the blue curve is the progression of infection. Single mutations may be eliminated for sufficiently strong immune responses. If the immune response is less strong, the cancer and infection will start growing. For small times, the growth of both cancer and infection is logistic and each approaches an equilibrium (grey region). Thereafter, the exhaustion of cancer specific cytotoxic T-cells is imposed at year 22 (stipulated curves in right white region), which enforces the cancer and infection to escape. The exhaustion is included by allowing active cytotoxic T-cells to become inactivated with an exhaustion rate. As a result, the three E’s of immunoediting follows from the model. The full curves before escape may not cause symptoms but the stipulated part of the curves will sooner or later result in diagnosable conditions as these will continue increasing toward a value near 1 (not shown). However, the degree of exhaustion determines the escape growth rate.

Thorough analysis shows that the parameter space divides into two qualitatively different situations. In the first case (Type I) a unique and stable co-existing steady state exists and, in the other case (Type II), two stable steady states exist (and one unstable steady state), simultaneously. Treatments affecting the parameter values may shift the situation from one case to the other. The disease burdens related to the steady states vary with the values of the parameters. The case of a single co-existing stable steady state has been reported and discussed elsewhere [19,54,55,56]. Thus, the present focus will be on the novel case of two co-existing stable steady states (bi-stability) and we will choose a suitable set of the parameter values denoted by the default parameter values, which will permit this case (see Appendix A). One of the two steady states has low cancer and infection burden, while the other has high cancer and infection burden; thus, we associate these with a dormant or pre-cancerous state and a full-blown disease state, respectively. A curve in the phase-space has cancer burden on the first axis and infection burden on the second axis and separate points are attracted toward the dormant state from those that are attracted toward the full-blown cancer-infection state. This separatrix divides the phase plane of feasible states into two regions, the basin of attraction for the dormant state and the basin of attraction for the full-blown disease state, as illustrated in Figure 3. The exhaustive numerical examinations show that high levels of either infection or cancer in the dormant state are rare. Likewise, low levels of infection or cancer in the full-blown disease state are rare and extreme parameter values are needed to be realized (see the sensitivity analysis in Appendix A). The following observations hold for the generic Type II case represented by our default parameter values. All non-boundary states, i.e., states with non-vanishing infection and malignant cells, are attracted toward either of the two co-existing stable steady states over time.

### 3.2. The Three E’s of Immunoediting Is a Consequence of the Cancer-Infection-Immune Response

The three E’s of immunoediting are elimination, equilibrium and escape follow from the model. The model predicts elimination of the mutations if the adaptive immune system is sufficiently strong, e.g., if α is large corresponding to $A_{1}$ and $B_{1}$ being large. If not, a logistic growth will initiate, resulting in an equilibrium. The equilibrium is a saturation level and in the case of bi-stability it is interpreted as a dormant state also denoted by a pre-cancerous state. The exact level of infection and malignant cells in such dormant state depends on the values of the parameters. If a sufficient amount of infection or cancer is suddenly added, the system may progress into full-blown cancer, see Figure 3. Another possibility is that some parameters may be perturbed such that the dormant equilibrium disappears and the only stable steady state left is the full-blown cancer-infection state, which then will be globally stabilize in the open half-plane, meaning that all co-existing states will approach the full blow cancer-infection state over time (see below). This is identified as the escape phase, where the infection and cancer are growing toward a new equilibrium of high cancer and infection.

### 3.3. Infection Trigger Cancer Escape from Immune Surveillance

**Figure 4:**
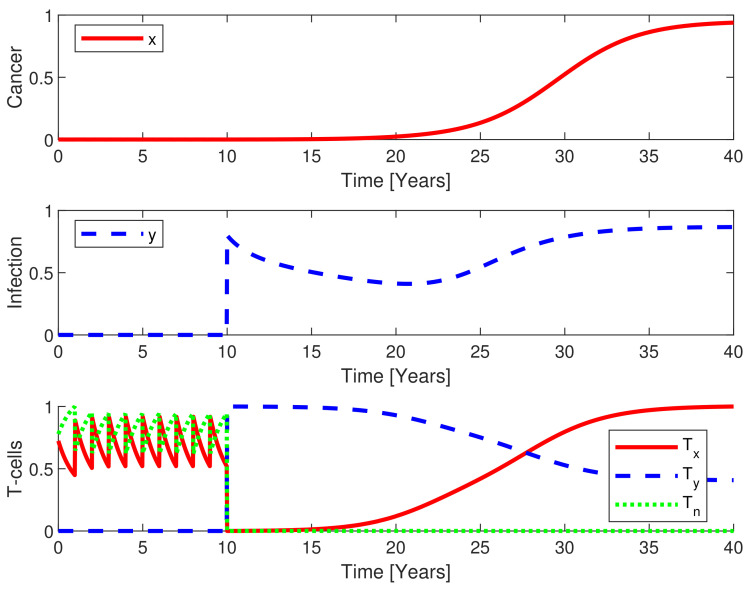
Despite an annual malignant mutation, the cancer is eradicated or kept in a dormant state (upper panel) for the first ten years by immunosurveillance in the absence of infection. At year 10, a severe infection is imposed (middle panel) and, thereafter, malignant mutation blooms into a cancerous clone capable of growing exponentially for a while whereas the development at the later stage is logistic (lower panel). This is caused by the infection taking resources from the immune system in fighting the malignant mutations. Whenever the cancer grows in size, it takes further resources from the immune system. Thus, the infection assists the cancer escape and whenever the immune system fails to eliminate the cancer, the infection grows too. This is a self-promoting spiral accelerating both the cancer progression and infection.

Consider a case where a single cancer mutation occurs each year (see Figure 4). The immunosurveillance may eradicate these mutations through the innate immune response. If this response is insufficient the adaptive immune system will attempt to suppress the amount of malignant cells. Thus, the state will return to the dormant state in the absence of a severe infection. Adding an infection during suppression of a low cancer load may result in cancer escape. This is due to the allocation of active immune cells for defeating the infection, thereby taxing the immune system and limiting the activation of cancer specific effector cells that are fighting the cancer. Thus, the immune system becomes increasingly challenged as the cancer grows in size, see Figure 4.

If the infection is raised abruptly in the model, then the immune system responds by activating infection specific effector cells to deal with the infection. The larger the amount of additional infection, the larger the response by the immune system. Such response results in a proportional reduction in cancer specific effector cells causing the cancer to escape by growing exponentially. The separatrix constitutes a ‘threshold’ below which the infection is defeated by the adaptive immune system and above which it escapes the immunoediting and cancer progresses. Hence, this separatrix separates cancer-infection progression and self-recovery. In the case of low additional infection, the immune system is capable of combating the infection while the cancer grows slightly before returning to the dormant state level. In the case of large additional infections, the immune system is not capable of defeating the infection sufficiently fast while the cancer increases above the escape level. In the case of escape, the infection declines for some time while the cancer grows. The immune system tries to defeat the cancer but the increasing cancer level requires increasing resources from the immune system in an attempt to win the battle. At a certain instant, the infection escapes the immune system and, simultaneously, the cancer blows up. The outcome depends crucially on which side of the separatrix the state is moved to at the time period right after the additional infection has been imposed.

### 3.4. Exhaustion of Cancer Specific Cytotoxic T-Cells Causes Cancer Escape

The adaptive immune system reacts to pathogens by activating pathogen specific cytotoxic T-cells and a cascade of cytokines, chemokines, etc., result. After a while, the regulatory T-cells dampen the inflammatory response if the pathogen is successfully downregulated. However, if the inflammation is not controlled, it may result in chronic inflammatory diseases and the exhaustion of the cytotoxic T-cells. Such exhaustion may be imposed in the model as active cancer specific cytotoxic T-cells (CD8+) become inactivated (CD8) after certain exposure, e.g., as the accumulated response of regulatory T-cells reaches a certain threshold level. In the model, we imposed an exhaustion effect by inactivating cytotoxic T-cells by a first order depletion rate after a period of exposure. As a result, the three E’s of immunoediting follows as an intrinsic feature of the model. After elimination for strong immune responses or a growth phase resulting in temporary equilibrium, an additional exhaustion mechanism or inactivation of cancer specific effector T-cells may allow the cancer to escape towards full-blown cancer if it is not treated afterward, as illustrated in Figure 3. To illustrate this effect, a dysregulated additional exhaustion of the cytotoxic T-cells is initiated at year 22 and the increase in the resulting plateau of the equilibrium is studied. If the exhaustion rate is relatively low, e.g., 9.2 times the natural death rate ($d_{x}$) and starts at year 22, the 50% allele burden appears at year 65. If the exhaustion rate is slightly lower, e.g., nine times the natural death rate, the escape takes place much later and the 50% allele burden appears at year 110. By increasing the exhaustion rate, e.g., to 10.5 times the natural death rate, the 50% allele burden appears earlier, e.g., at year 48. Thus, the growth of the escape is very sensitive to the exhaustion rate but in all cases the cancer escapes and ultimately results in full-blown cancer; the cancer progression is then followed closely by a corresponding progression in infection. For exhaustion rates smaller than nine times the natural death rate, the escape happens after an average human lifetime for our default parameter values.

### 3.5. Early Treatment of Infection Prevents Cancer Progression

**Figure 5:**
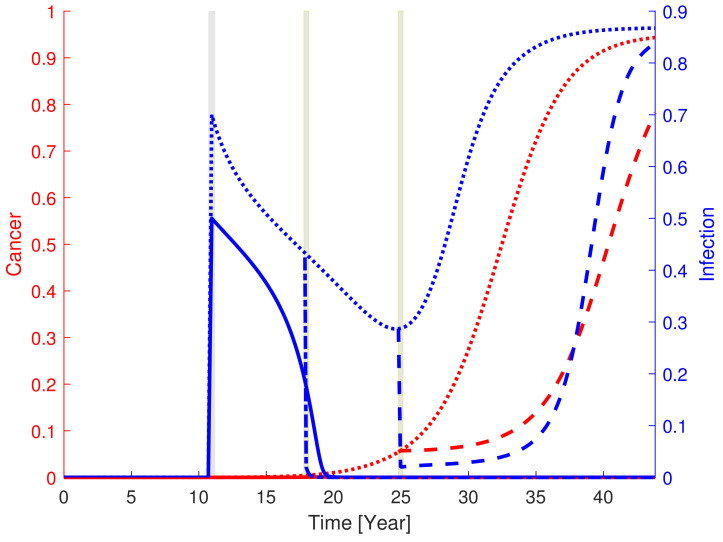
Progression of cancer (red) and infection (blue). Model response to a sudden additional infection introduced at year 11 (left grey region). A threshold value for the additional infection exists, which separates cancer-infection progression (dotted curves) and recovery (full curves). If modest infection is added, the immune system is capable of defeating the infection (full blue curve) while the cancer only blooms slightly before eventually returning to the dormant state level. In the case of high infection, the immune system is not capable of defeating the infection sufficiently fast (dotted curves). The infection is lowered for some time while the cancer grows (dotted curves). However, if the infection is treated sufficiently fast (middle grey region), e.g., 7 days after infection onset (dash dotted curves), then the state comes below the separatrix and the immune system is capable of defeating the growing amount of malignant cells as well as the infection. Treating infection downwards (right grey region/stipulated curves) may be insufficient and result in infection-cancer escape.

A dormant state with small cancer load can result in escape upon obtaining a severe or sustainable infection and, subsequently, full-blown cancer progression as depicted in Figure 4. This observation makes it tempting to perform an in silico investigation of how such a situation is affected by antibiotics. Presuming that a significant infection (moving the state to above the separatrix) is imposed at year 11, the cancer starts to progress exponentially, see Figure 5. In the absence of treatment, the cancer develops into full-blown cancer accompanied by a high level of infection. In this case, it is tempting to conclude that the infection drives the cancer progression. However, the dynamics are a common phenomena. The cause-action is not uni-directed and the two pathogens affect each other in a circular manner. Treatment suppressing the infection to a modest level (moving the state to below the separatrix) at year 18 reverses the development and the cancer and infection returns to the dormant state. Henceforth, the immune response controls the disease. However, if the treatment is instead initiated later, at year 25, reducing the infection to the same level as in the preceding case while the cancer has developed (resulting in a state above the separatrix) then a temporary decrease in cancer is observed but ultimately a relapse follows. The state approaches the full-blown cancer-infection state over time, see Figure 5.

### 3.6. CAR T-Cell Immunotherapy Shows Good Effect but Is Improved in Combination with Antibiotics

In the last decades, chimeric antigen receptor (CAR) T-cell therapy has developed as novel and promising immunotherapy [57]. T-cells extracted from patient blood are modified in vitro to express artificial receptors targeted to specific tumor antigen.

In the following, we simply assume CAR T-cell therapy affects the virtual patient by adding an additional amount of effective T-cells to the cancer specific effector pool per time.

However, the mechanisms are different from that of normal T-cells, since CAR T-cells identify the tumor antigen without involving the major histocompatibility complex. Despite this fact, we denote the ‘strength’ of the CAR T-cell therapy in percentage in terms of the normal cytotoxic T-cell response, i.e., the CAR T-cell dosage refers to the effect it causes in percent of effect of the otherwise normal cancer effector T-cell response.

**Figure 6:**
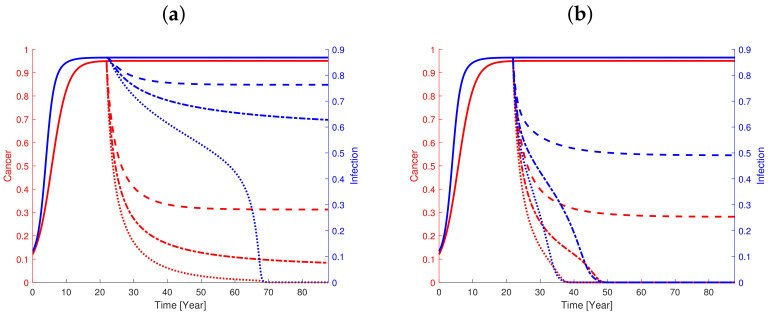
The progression of cancer (red curves and left axis) and infection (blue curves and right axis) for default parameter values upon escape (full curves). (**a**) At year 22, a virtual patient with full-blown cancer-infection is exposed to CAR T-cell therapy by adding modified cancer specific effector T-cells. By adding 60% and 80% CAR T-cell of the otherwise normal response (stipulated curves and dash-dotted curves, respectively), cancer and infection are reduced but not normalized. Increasing the dosage to 100% causes the virtual patient to be cured but after an insufficiently long period of time (50 years). Cancer burden decays ‘exponentially’ while infection shows a pronounced shoulder in the response before continuing the fast decay. Ultimately the state approaches the dormant state. If treatment is stopped before the infection level becomes sufficient low, the virtual patient experiences a relapse (not shown). If treatment is stopped after both cancer and infection have been lowered to the neighborhood of the dormant state, no relapse is observed. (**b**) Similar to left panel but, in addition to CAR T-cell therapy, there is also an anti-inflammatory treatment provided with similar dosages. These combi-treatments result in significantly improved effects of the in silico treatments compared to the corresponding CAR T-cell mono-therapy. For the 80% dosage, the combination treatment becomes successful. For the largest dosage of 100%, the treatment time to reach success is reduced from 50 years to 12 years and the time to cure is halved compared to the combination treatment with 90% dosage.

In the model the CAR T-cell therapy is simulated by adding an additional source to the right hand side of Equation (4). The in silico effect of such immunotherapy on cancer-infection progression for default parameter values after an escape is examined, see Figure 6. For large dosages of CAR T-cells (100% above normal), the cancer burden initially decays relative rapidly but ends with a prolonged slow decay, while infection demonstrated a delayed shoulder response before decaying.

For the virtual response to CAR T-cell therapy (see Figure 6) the infection level first declines fast, while the cancer load declines faster. For the infection, a temporal shoulder is noticed, which has a delayed impact on cancer reduction. When the cancer level is very low, the decline in infection becomes fast. Ultimately, the state approaches the dormant state. Over time, the natural immunoediting takes over and controls the state to remain in a narrow neighbourhood of the dormant state if not further perturbed. As for other treatments, a threshold for sufficient treatment dose must be crossed to approach the dormant state. If treatment is stopped prematurely, the virtual patient shows a relapse (not shown). This is in contrast to ending the treatment when the cancer-infection state has reached the basin of attraction for the dormant state where no relapse appears. For even stronger dosages of CAR T-cells, the cancerous decay is faster and the duration of the shoulder becomes much shorter. Decreasing the dosage (e.g., to 80% above normal) significantly amplifies the shoulder phenomena and treatment has only partial effect during a person’s lifetime. For even smaller dosage of CAR T-cells (e.g., 60% above the normal) the cancer-infection does not cross the separatrix in due time as in the case of 80% dosage, but an intermediate plateau in the basin of attraction for the full-blown cancer-infection state is reached. Treatment cessation will cause a rapid relapse in this case.

The size of sufficient dosage depends on the actual individual parameter values and the amount of cancer when the treatment start. If in addition to CAR T-cell therapy, an anti-inflammatory treatment is also given. The treatment period is substantially reduced compared to the CAR T-cell mono-therapy. The in silico combination treatment results in a significantly improved effect compared to the CAR T-cell mono-therapy. For the 80% dosage, the combination treatment is successful. For the largest dosage of 100%, the treatment time to reach success is reduced from 50 years to 12 years and the time halves compared to the combination treatment with 90% dosage.

However, the infusion of CAR T-cells may cause the patient’s immune system to react by diminishing the otherwise natural T-cell formation as a response to the cancer. In the model, we imposed such combined treatment with inhibitory feedback on the natural T-cell production by adding CAR T-cells while simultaneously lowering the natural production of T-cells in response to the cancer. Specifically, this is performed by decreasing either the rate by which cancer stimulates the adaptive immune system to activate cancer specific effector cells ($\beta_{x}$) or the baseline production rate of the T-cell regulatory pathway (α), or both. In the case where only α is reduced to, e.g., 80%, the CAR T-cell therapy only results in a partial effect over a life-time. The cancer reduces by approximately 25% while the infection is reduced by 10% or less. For 90% and 100% CAR T-cell in combination with anti-inflammatory treatment, the time to remission is approximately doubled. From simulations, it follows that the cancer reduction is much more pronounced if the infection is reduced too. This is because the cancer burden declines faster whenever the infection declines fast. While $\beta_{x}$ has some reducing effect on infection, α does not affect the infection noticeably. The case where both α and $\beta_{x}$ are reduced to, e.g., 90% is very similar to the situation where $\beta_{x}$ is changed but the dynamics are slower. Here, the treatment needs to be prolonged by 25% to reach remission. If α or β or both are reduced further, the treatment effect becomes less effective. Thus, a patient whose immune system counteracts the therapy may show poor response to the CAR T-cell therapy.

In conclusion, in order to force the cancer to a neighborhood of the dormant state in order to obtain a permanent effect on the virtual patient, the CAR T-cell therapy should be sufficiently strong and the treatment last sufficiently long. The effect of CAR T-cell treatment is significantly improved when combined with antibiotics.

## 4. Discussion

We have used mathematical modeling and in silico experiments to investigate the basic principles of the interaction of malignant and infected cells with the adaptive immune system. Pathogens stimulate the adaptive immune system to activate specific effector cells, i.e., the cancer and infection effector cells, to fight the respective pathogens. By presuming that these effector cells share common predecessors or more precisely regulated pathways, the two diseases are coupled to each other by the immune system. In situ, such pathways involve naïve T-cells as predecessors shared by the pathogen specific cytotoxic T-cells (but the limiting factor could be the antigen presenting cells or regulatory cytokines without changing the outcome of the simulations). Thus, these effector cells may compete in taking resources from one another, e.g., increasing infection may require more infection specific effector cells and thereby reduces the production of cancer specific effector cells and vice versa. This hypothesis is implemented in the mathematical model, which is the coupled cancer-infection-immune model, and the consequences are examined. The simple model explains many well-known evidence-based observations. These observations are reduced to consequences of the simple ‘theory’ constituted by the proposed model. Of course, this is an idealization of the reality and a cover for many subcomponents working together. Inter-variations and intra-variation and noise appear in the real world, but, when comprehensively interpreted, the model outcome and predictions are qualitatively robust to situations.

We apply the aforementioned quantitative approach and provide the criteria for sufficient immune surveillance or, contrarily, the escape of a malignant clone. The proposed model encapsulates and explains the dynamics behind the three E’s of immunoediting [5,58]. In the model, all cancer-infection states are generally either attracted toward a dormant state of low disease burden or toward a full-blown state with high disease burden. A separatrix is separating regions of states attracted toward the two states. Mutations may happen frequently in the modeling framework but in rare cases, e.g., of severe infection, they may escape the immune surveillance and grow into diagnosable cancer. Related to this, in silico treatment of cancer may either be successfully interpreted as the malignant clones have been eradicated or at least were forced back into a dormant pre-cancerous state or were unsuccessful, i.e., the cancer may relapse after treatment ends. The *CALR* mutation frequently seen in MPNs may be eradicated corresponding to the number of malignant cells in the dormant state becoming smaller than one or at least so low that normal fluctuations in the innate immune response are capable of eradicating the malignancy. The proposed model suggests that this treatment may bring the allele burden below aforementioned separatrix and, thereafter, the immune system may eradicate the cancer. In contrast, the *JAK2*V617F mutation also seen in MPNs is harder to eradicate, which in the model is explained by an inappropriate low but not vanishing number of malignant cells in the dormant state.

In the present paper, we focus on advantages, possibilities and obstacles of immunotherapy [59]. We consider immunotherapy in silico, i.e., chimeric antigen receptor (CAR) T-cell therapy [36,37,60]. The model shows how the progression of cancer and the effect of treatments inherently depend on the coupling and the levels of cancer and infection. Therefore, the treatments are also investigated in combination with anti-inflammatory treatment. The bi-stability of the model is important for separating a dormant state and a full-blown cancer-infection state. The separatrix lies much closer to the dormant state than to the full-blown cancer-infection state. Thus, a relatively small perturbation at the dormant cancer-infection state may result in escape while a large perturbation is needed for reversing the disease progression.

Briefly three major conclusions arise in silico: Firstly, the treatment with a given dosage is vital for good response while a slightly later onset of treatment with similar dosages may be unsuccessful. Secondly, severe infections, e.g., by COVID-19 virus and by Morbus Crohn or maybe from obesity, aging and smoking, may result in cancer escape. Thirdly, sufficient large dosages are recommendable if not causing severe side effects and the treatment effects improve significantly in combination with antibiotics.

Our overall findings based on the model and by exhausting investigations of the possible values of the parameters may be summarized as follows:

1. Ongoing mutations may either be eradicated by the immunoediting, kept in low numbers in a dormant state or a malignant clone may escape the immunoediting and expand which results in diagnosable cancer that will progress toward full-blown cancer if left untreated. The dormant state may be thought of as a potentially pre-cancerous state, since malignant cells at low burden are rarely symptomatic. In hematopoietic cancers, such dormant states are referred to as clonal hematopoiesis of indeterminate potential (CHIP) and it requires an activation of the immune system by the malignant cells in order to control the cancer;
2. The model illustrates how tumor immunoediting explains the transitions between health and disease depending on the inflammatory load caused by non-cancerous infectious factors. Such inflammation could include chronic inflammation, e.g., caused by inflammatory bowel diseases and severe virus infections such as COVID-19 or even obesity, aging and smoking;
3. The model explains how an infection may compromise the immunosurveillance controlling the cancer as they are sharing pathways of the immune system. In particular, a severe infection or T-cell exhaustion may result in cancer escape;
4. The model explains which pathophysiology, e.g., which disturbances of the common integrated system, results in cancer progression and which of these are ‘easily’ reversed or are harder to reverse by immunotherapies;
5. In accordance with evidence-based knowledge, most patients show relapse after treatment is paused, e.g., in *JAK2*V617F-positive MPNs, while the treatment result may last in *CALR*-positive MPNs;
6. In silico investigation of CAR T-cell therapy implies that strong and sufficient persistent immunotherapy may last;
7. Sufficient early and strong treatment with CAR T-cell therapy shows good response for the virtual patient while postponed treatment may fail;
8. Combining CAR T-cell therapy with immune-modulating antibiotic improves the effect of the treatment significantly and, in some cases, renders unsuccessful treatments successful;
9. The model confirms the evidence-based experiences described by the “three E’s of immunoediting”, elimination, equilibrium and escape.

The conclusions must be taken with some precaution since these depend on the decreasing potential of hypothesis [35]. In order to reduce the huge complexity of the immune system, we have lumped all the inhibitory feedback mechanisms regulating the activation of pathogen specific effector T-cells into one mechanism, the T-cell regulatory pathway. Thus, we have represented the T-cell regulatory pathway by a single lumped compartment, with a limited production rate. This approach hides many details and blurs the contribution of the individual mechanisms. The choice to keep the production rate α constant is conservative and it represents a limiting bottleneck for the instantaneous capacity of activating pathogen specific effector T-cells. We emphasize that the lumped nature of the T-cell regulatory pathway may render both α and ϵ dependent on the specific pathogens considered. Nevertheless, the model proposes an idea for how the immune system bridges different cancers and cancer associated infectious diseases, which may be the common unifying explanation for the clinical observations listed above.

## Figures and Tables

**Table 1:** The parameters in Equation (6) expressed in terms of the original parameters and their nominal values.

$A_{1}$	$A_{2}$	$A_{3}$	$A_{4}$	$B_{0}$	$B_{1}$	$B_{4}$
$\frac{\alpha r_{x}p_{x}}{a_{x}d_{x}}A_{2}$	$\frac{\beta_{x}K_{x}}{\epsilon }$	$\frac{\beta_{y}K_{y}}{\epsilon }$	$\frac{r_{x}K_{x}\left(1-p_{x}\right)}{d_{x}}$	$\frac{a_{y}}{a_{x}}$	$\frac{\alpha r_{y}p_{y}}{a_{x}d_{y}}A_{3}$	$\frac{r_{y}K_{y}\left(1-p_{y}\right)}{d_{y}}$
$5.7\times 10^{4}$	$7.5\times 10^{3}$	$5.6\times 10^{3}$	$1\times 10^{3}$	3.04	$1.01\times 10^{5}$	20

**Figure A1:**
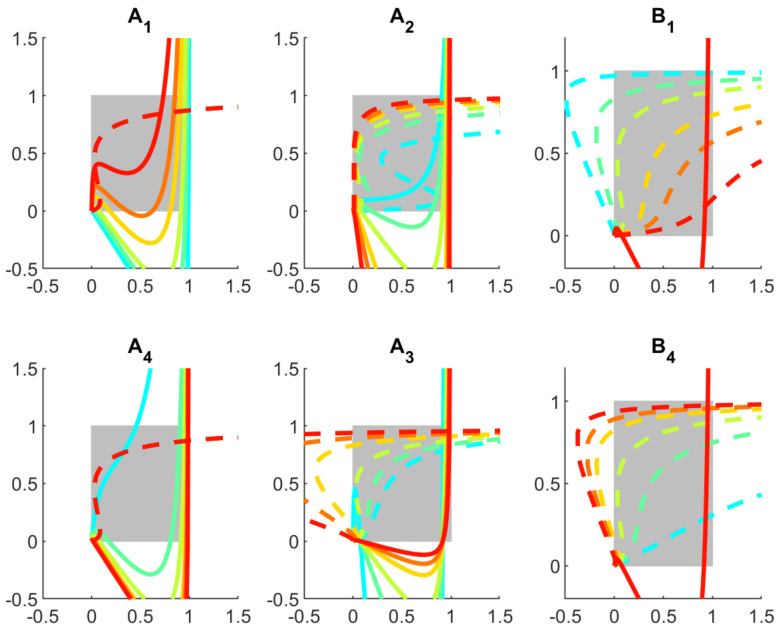
By modest variation in the individual parameter values by factors 0.1, 0.5, 1, 2, 3 and 5 of their default values, the effects on the nullclines and the steady states are observed. The grey region illustrates the physiological feasible range of the variables while a broader range is shown to emphasize the nullclines. Pairwise nullclines have the same color code while the *X* nullclines are full curves and the *Y* nullclines are stipulated.

**Figure A2:**
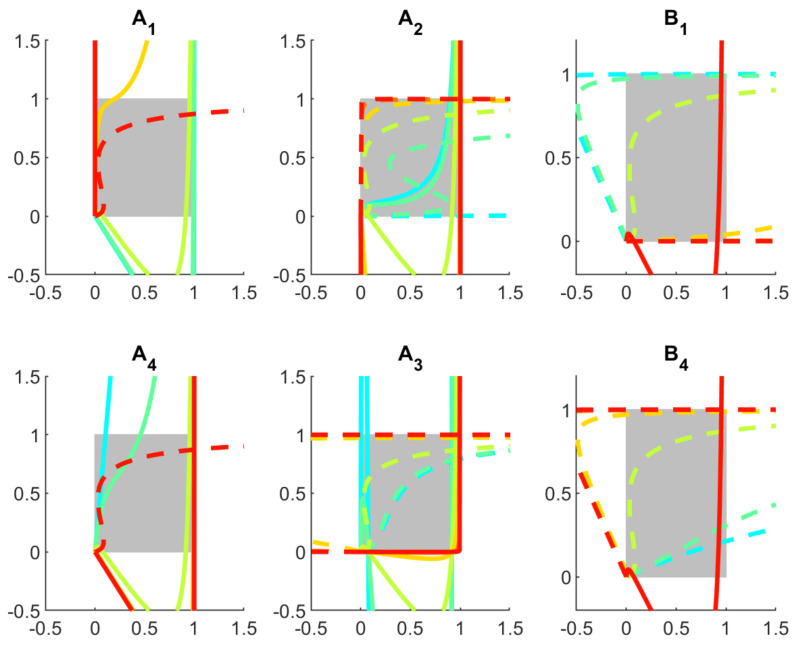
By larger variation in the individual parameter values by factors $10^{-2},10^{-1},1,10^{1},10^{2}$ and $10^{3}$ of their default values, the effects on the nullclines and the steady states are observed. Pairwise nulclines have the same color code while the *X* nullclines are full curves and the *Y* nullclines are stipulated while the grey square represents the physiologically feasible states. Note that the pairwise nullclines at most have three intersections whereof at most two correspond to stable steady states.

## Data Availability

Not applicable.

## Appendix A

### Appendix A.1. Parameter Values

Despite large variation in parameter values between different cancers, the dynamics of the system is robust. Two different dynamical topologies are possible corresponding to the existence of either one or two co-existing stable steady sates. Thus, the specific values are illustrative but should be taken with care. In the sequential, we extract parameter values for JAK2 positive Philadelphia negative MPNs and HIV-1 infection from literature.

In cancers, we estimated $a_{x}$ to $1\times 10^{-3}$ per day [61], which is of the same order as in cancer medicine [20], where we estimated $a_{x}$ from three individual low cancer burden in vivo data sets to be in the range 0.2–8.5 cell per year. This corresponds to $0.5\times 10^{-3}$–$2\times 10^{-2}$ per day. Thus, we choose $a_{x}=1.25\times 10^{-3}$ per day, which is slow compared to most other cancers (for which $a_{x}$ lies between 0.01–1.5 per day [29,34,55,59,62,63,64,65,66,67,68]).

In [19,69], the carrying capacity, $K_{x}$, is estimated indirectly to be $10^{4}$ while [70] used $2\times 10^{4}$. We chose $K_{x}=1.2\times 10^{4}$, which agreed well with measurements of the number of hematopoietic stem cells [71] and our earlier estimate $3\times 10^{4}$ in [55].

Across various cancers, the values of the product $r_{x}\cdot p_{x}$ varies ranging from $5\times 10^{-11}$–0.5 per day [34,62,63,64,65,66,68]. Kuznetsov [28,29] took a value for $p_{x}$ near to but smaller than 1, e.g., 0.998, for BCL1 lymphoma in non-chimeric mice based on data from [72]. For leukemia, $r_{x}p_{x}=0.002$ per capita per day are used in [19,70]. We choose to use a slightly smaller value $p_{x}=0.8565$ than Kuznetsov did which corresponded to $r_{x}=2.3233\times 10^{-2}$ per capita per day. Thus, our value for $r_{x}\left(1-p_{x}\right)$ becomes $1.9900\times 10^{-3}$ per capita per day.

Natural death rates for active cytotoxic T-cells are reported to be in the range $4.12\times 10^{-2}$–0.12 per day [62,64,65,66,73]. We chose $d_{x}=0.4$ per day.

The initial exponential growth has been carefully fitted to the data in [74], resulting in the value for $\beta_{x}=0.18$ per capita per day. However, [75] estimate $\beta_{x}$ by optimal control to around 0.02 per capita per day while [76,77] report $\beta_{x}$ to be around $4\times 10^{-4}$ per capita per day. We choose $\beta_{x}=0.125$ per capita per day.

The baseline production rate α is reported as high than 225 cells per day [78], but in [68] it is estimated to be 0.33 per day, while [19] found 0.19 per day. We chose α=0.5025 per day as a compromise.

In [65], the natural death rate for naïve T-cells is measured at $2.4\times 10^{-2}$ per day. We chose $\epsilon =2\times 10^{-2}$ per day as our default value, which is also in agreement with [79] who have measured it to be in the range 0.01–0.1 per day. We used these values to represent those of the T-cell regulatory pathway.

Particular infectious agents are all different, some develop fast, e.g., bacterial infections such as those involved in Sepsis [80] or some viruses, e.g., acute HIV-1 infection [81], whereas other are slow. The growth rate for acute HIV-1 infection measured in plasma is estimated to be between 0.37 and 2.7 per day in [81]. Slow viruses have long incubation periods ranging from months to years and are caused by slow replication rates [82]. Examples of slow viruses encompass Measles virus (1–10 years) [83], Rubella virus (10–20 years) [83], Rabies virus (3–12 weeks) [84], JC virus (years-life) and BK virus (year–life), whereof the last two are observed in immunosuppressed patients only [83]. Since we mainly consider slow infections, we decided on $a_{y}=3.8\times 10^{-3}$ per day.

We use a modest value for the carrying capacity $K_{y}=1200$, where [85] has a peak value around $10^{9}$ cells.

In [85], $\beta_{y}$ is measured to be 0.96 per capita per day, while [86] has 0.52 per capita per day. We chose our $\beta_{y}=0.933$ per capita.

In addition, [85,86] have measured the natural death rate $d_{y}$ to be 0.6 and 0.65 per day, respectively. We have chosen $d_{y}=0.4$ per day in agreement with that for $d_{x}$.

The authors of [86] have measured $r_{y}\cdot p_{y}$ to $5.74\times 10^{-4}$ per capita per day while [85] measured it to be a factor of 100 less. Thus, we chose $r_{y}\cdot p_{y}=3.7\times 10^{-4}$ per capita per day. We have no source on $r_{y}$ and $p_{y}$ individually but chose these to be approximately $r_{x}$ and $p_{x}$, respectively, and at the same time they possess the right product. Hence, we chose $r_{y}=5.198\times 10^{-3}$ and $p_{y}=0.7178$. These choices provides $r_{y}\cdot \left(1-p_{y}\right)=1.5\times 10^{-4}$ per capita per day.

### Appendix A.2. Sensitivity Analysis

We emphasize that the analysis of the model has qualitatively two types of dynamics. These are determined by the dimensionless parameter values, which are clusters of the physiological parameter. More precisely, the dimensionless parameters depend, e.g., on the fractions $\beta_{x}/\epsilon$ (for $A_{2}$), $\beta_{y}/\epsilon$ (for $A_{3}$), $r_{x}/d_{x}$ (for $A_{1}$ and $A_{4}$), $a_{y}/a_{x}$ (for $B_{0}$), $r_{y}/d_{y}$ (for $B_{1}$ and $B_{4}$) and $a_{x}/\alpha$ and $a_{y}/\alpha$ (for $A_{1}$ and $B_{1}$) but not on the individual values of these physiological parameters (see Table 1 in main text).

The dynamics of the system is completely determined by the nullclines as the intersections of these determine the steady states. Below, we show how the nullclines and their intersections vary with the parameters with an onset in the default values found above. Varying the parameter values changes the number of stable steady states and their exact location in $R_{+}^{2}$. As mentioned, the number of stable steady states are either one or two. We vary the parameter values by factors 0.1, 0.5, 1, 2, 3 and 5 of their default values and observe the effect on the nullclines, see Figure A1 and by factors $10^{-2},10^{-1},1,10^{1},10^{2}$ and $10^{3}$ in Figure A2. Notice that $A_{1}$ and $A_{4}$ only affect the nullcline of *X* by lowering the curves for increasing parameter values and, similarly, $B_{1}$ and $B_{4}$ only affect the nullcline of *Y* by moving these leftward with increasing parameter values, while $A_{2}$ and $A_{3}$ affect both nullclines by moving the *X* nullclines downward and moving the *Y* nullclines leftward. The steady state values become more extreme as the full-blown state approaches (1,1), while the dormant state approaches (0,0). Similarly, the unstable steady state approaches (0,0) with increasing parameter values. Before reaching (0,0) the dormant state fuses with the unstable steady state and both disappear in a bifurcation.

### Appendix A.3. Quasi-Steady State Approximation

The time scale for cancer growth is $a_{x}=1.25\times 10^{-3}$ per day while that of infection is $a_{y}=3.8\times 10^{-3}$ per day. Likewise, the time scale for cytokines, naïve cancer specific cytotoxic and infection specific cytotoxic T-cells are α=0.5025 per day, $\beta_{x}K_{x}=1.5\times 10^{3}$ per day and $\beta_{y}K_{y}=1.12\times 10^{3}$ per day, respectively. Thus, the fraction of the time scales between cancer and infection development versus immune cell growth is $10^{6}$. Being interested in the long term development, the transient dynamics of the immune system are fast. Thus, this part denotes the fast manifold behavior as it enters into ‘equilibrium’ instantaneously compared to the slower part of the system (the slow manifold). Hence, the slow manifold approximation follows from requiring the derivatives of $T_{n}$, $T_{x}$ and $T_{y}$ to be zero. For further interest in such model reductions, see [87].
